# Lipid-mRNA nanoparticles landscape for cancer therapy

**DOI:** 10.3389/fbioe.2022.1053197

**Published:** 2022-10-31

**Authors:** Yin Li, Hengtong Fang, Tao Zhang, Yu Wang, Tingting Qi, Bai Li, Huping Jiao

**Affiliations:** ^1^ College of Animal Science, Jilin University, Changchun, Jilin, China; ^2^ Department of Colorectal and Anal Surgery, General Surgery Center, First Hospital of Jilin University, Changchun, Jilin, China

**Keywords:** mRNA delivery, lipid nanoparticles (LNPs), ionizable lipids, cancer nano-vaccine, cancer immunotherapy, gene editing

## Abstract

Intracellular delivery of message RNA (mRNA) technique has ushered in a hopeful era with the successive authorization of two mRNA vaccines for the Coronavirus disease-19 (COVID-19) pandemic. A wide range of clinical studies are proceeding and will be initiated in the foreseeable future to treat and prevent cancers. However, efficient and non-toxic delivery of therapeutic mRNAs maintains the key limited step for their widespread applications in human beings. mRNA delivery systems are in urgent demand to resolve this difficulty. Recently lipid nanoparticles (LNPs) vehicles have prospered as powerful mRNA delivery tools, enabling their potential applications in malignant tumors *via* cancer immunotherapy and CRISPR/Cas9-based gene editing technique. This review discusses formulation components of mRNA-LNPs, summarizes the latest findings of mRNA cancer therapy, highlights challenges, and offers directions for more effective nanotherapeutics for cancer patients.

## Introduction

**FIGURE 1:**
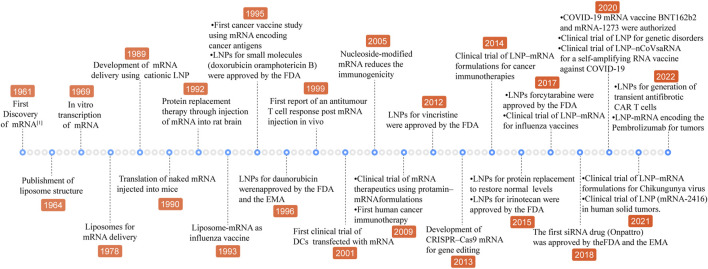
Hallmarks of mRNA-lipid nanoparticles. LNP, lipid nanoparticle; COVID-19, coronavirus disease 2019; CRISPR/Cas9, Clustered Regularly Interspaced Short Palindromic Repeats/CRISPR-associated protein 9.

Messenger RNA (mRNA) is an unstable intermediate in the central dogma conveying genetic information from genes to proteins. As the therapeutic agent, compared with DNA, mRNA is safe and has no potential risk of integrating into the genome. Additionally, the mRNA is not needed to transfer into nucleus, which is relatively easy for transferring and translation. In 1990, it was proved that *in vitro* transcribed (IVT) naked mRNA is translated in mice by direct intramuscular injection (Wolff et al., 1990). However, mRNA is unstable in harsh physiological conditions and is rapidly degraded by endoribonucleases and exoribonucleases of the extracellular and intracellular milieu before translating an encoded therapeutic protein (Dirisala et al., 2019). To overcome these shortcomings, the first thing is to improve the stability of mRNA. The mature mRNA molecules consist of five key elements, 5′ cap structure, 5′ untranslated regions (UTRs), coding regions, 3′ untranslated regions (UTRs) and 3′ poly(A) tail. The sequence of modification and optimization strategies have been built up for the above structural elements to augment translation efficiency and stability and to become immune-silent including modification mRNA with cap structure analog, non-nature base substitution and polyA tail synthesis (Kaczmarek et al., 2021). On the other hand, the delivery system remains another limiting factor for the employment of mRNA therapy. Subsequently, non-viral nanoparticles, such as polymer- and lipid-based materials were applied to cancer therapy (Cheng et al., 2018a; Cheng et al., 2018b). However, lipid-based nanoparticles show the advantages of providing protection for mRNA from nuclease degradation, accelerating acidic endosome escape after introduction into the cell and high payloads over polymer (Liu et al., 2021) and advances have been achieved in LNP-mRNA therapy (Figure 1). Lipids, which are the basic fabric of cell membranes, could self-assemble into nanoparticles in polar solvents and are essential for performing their biological functions. LNPs have excellent physical and chemical properties including their shape, size, surface characteristics and interaction with anionic molecules, thus they are widely applied in the medicine and health field as a delivery carrier for active pharmaceutical ingredients (Ndebele et al., 2022). Since LNPs have been used to deliver mRNA, a series of cancer nanomedicine was rapidly developed and entered into clinical trials, which made mRNA molecules possible to become a potent therapeutic system for cancer treatment (Kazemian et al., 2022).

In this Review, we focus on the mRNA modification and the key component of lipid nanoparticles in the process of formulation improvement and optimization. Subsequently, we highlight the progress in cancer therapy fields and describe key steps in tumor treatment. Finally, we discuss the future perspectives toward developing mRNA-LNPs as next-generation therapy vehicles.

## mRNA modification

### Cap structure analog design

**FIGURE 2:**
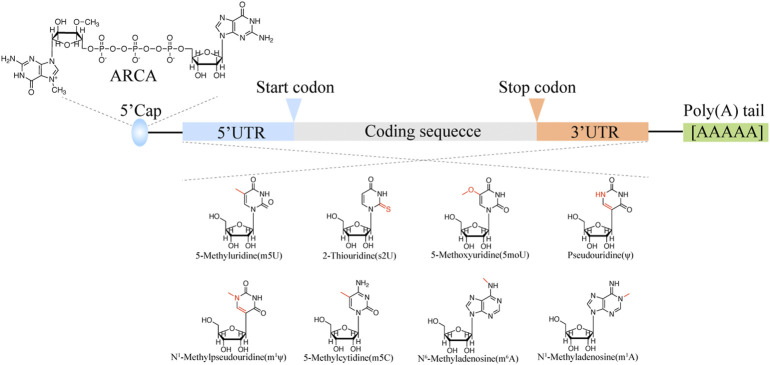
The optimization of IVT mRNA stability.

It is believed that mRNA lifespan is controlled by the 5′ cap structure and length of poly (A) tail. The mRNA is capped with the first base covalently linked with N^7^-methylated guanosine group *via* a 5′-5′ triphosphate bond (Hâkansson Wigley, 1998). *In vivo*, the RNA guanine-7-methyltransferase could cap the mRNA with GTP and Ado-met as the substrate. However, IVT RNA cap is inefficient. Both RNA polymerase and RNA ligase could ligate the cap structure (m^7^G(5′)ppp (5′)G) to the IVT mRNA 5′ terminals. Since the cap structure has two 3′ OH, almost half cap structure is reversed cap, that is Gpppm^7^GpN cap, which cannot be recognized by translation machinery (Carberry et al., 1990). Hence, the “anti-reverse” cap analogs (ARCAs) were designed to eliminate the possibility of reverse ligation of cap structure, which could get 2.7-fold protein translation efficiency than normal m^7^GpppG cap substrate (Figure 2).

### mRNA body modification

The mRNA body modification can also enhance translation efficiency and stability. The first found mRNA modification is m^6^A which is enriched in 5 termini of mRNA body. Inosine (I) is the intermediate of adenosine and guanosine, which is the nature modification found in the untranslated region. It is believed that inosine is a modulator for mRNA stability and translation efficiency. Pseudouridine also abounds in the mRNA coding region, which could increase levels of ribosome loading onto transcripts (Figure 2).

## Representative lipid component materials

**FIGURE 3:**
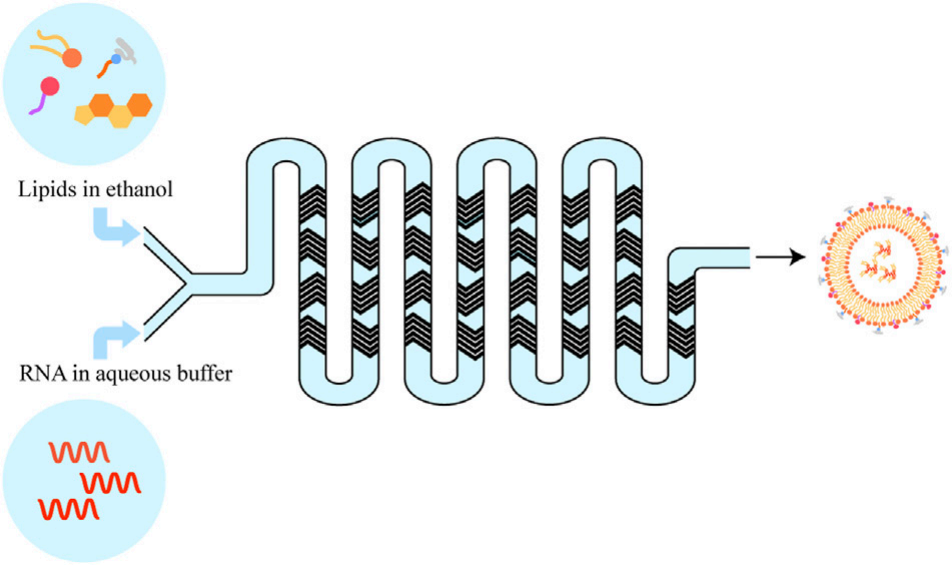
Scheme of next-generation lipid nanoparticles preparation.

Over the past several decades, the groundwork for genetic drug delivery platforms was laid. With the approval of two COVID-19 mRNA vaccines (Comirnaty^®^ (Pfizer-BioNTech), and Spikevax^®^(Moderna)), the preferred nano-delivery system is commonly lipid nanoparticles (LNPs) (Guerin Thompson, 2021). LNP can be prepared with different techniques, such as conventional lipid Thin-Film Hydration subsequent Size-Reduction Techniques, Ethanol-Injection Method, and next-generation In-line T-junction mixing and microfluidic mixing which are all based on a rapid mixing self-assembly process, namely a spontaneous organization of individual lipid molecule into a nanostructured sphere driven by non-covalent interactions (Figure 3) (Shepherd et al., 2021). Microfluidic mixing is a promising new method for preparing large-scale lipid-based cargos. It is well-made and can be expanded from the research laboratory to good manufacturing practice (GMP) production preferred nowadays for use in clinical trials and commercial pharmaceuticals. Another advantage of the microfluidics method is the ability to control various parameters. LNP formulations typically consist of 1) an ionizable or cationic lipid, possessing a positive charged tertiary or quaternary amines head to interact with the mRNA macromolecular; 2) a helper lipid (e.g., DSPC) acting like the phospholipid in the hydrophobic membrane; 3) cholesterol providing stabilization of the LNP bilayer; and 4) an outer PEGylated-surface to give the nanoparticle a hydrophilic surface, lengthen the blood circulation time and reduce nonspecific protein absorption (Kowalski et al., 2019).

### Cationic and ionizable lipids

**FIGURE 4:**
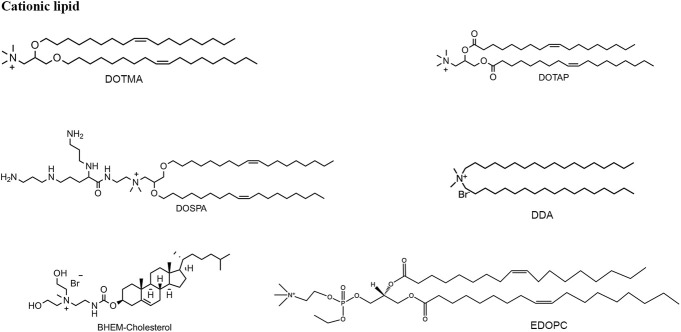
Structure of six typical cationic lipids.

**FIGURE 5:**
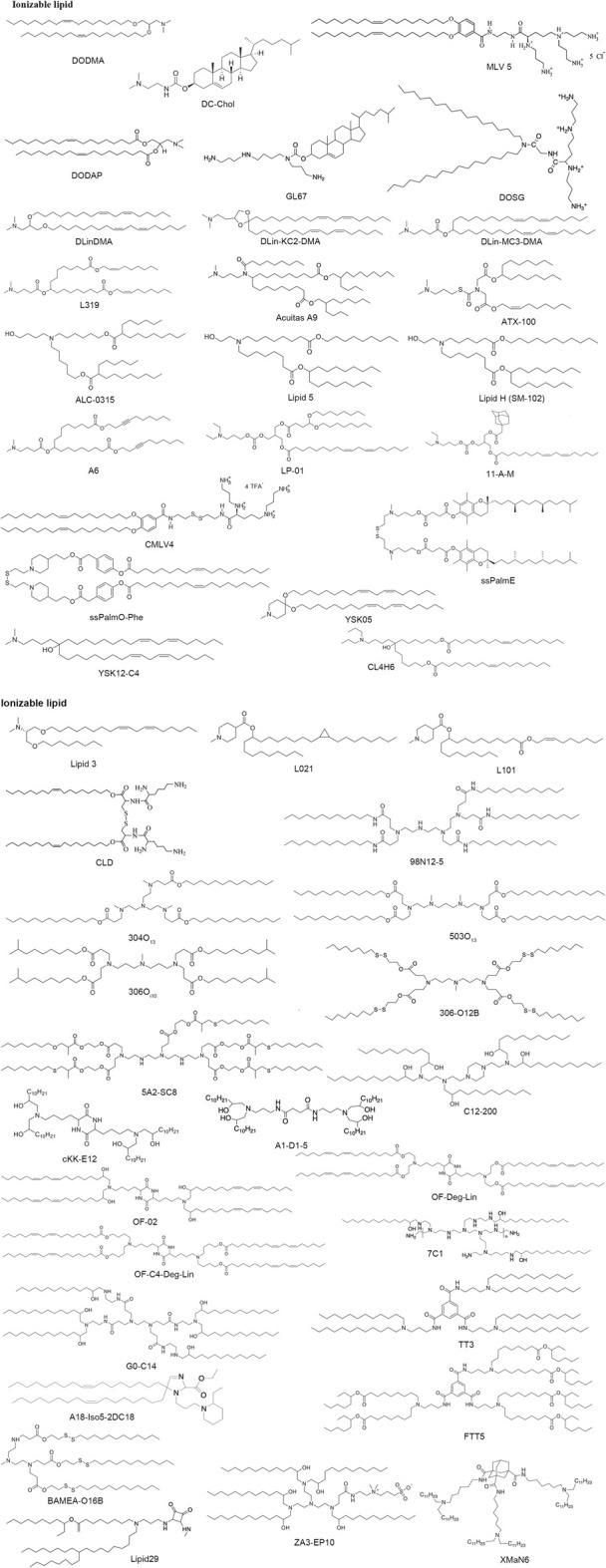
Structure of some typical ionizable lipids.,

Cationic lipids or ionizable lipids consist of three parts: A hydrophilic amine head region, a nonpolar alkyl tail group, and a core linker connecting the two domains (Figures 4, 5), acting as a capable nanomaterial to increase the entrapment efficiency. Cationic lipids bear nonionic ammonium head groups and remain in their pH-independent positive charge, such as DOTMA, DOTAP, DOSPA, and EDOPC. The mRNA-lipoplexes (RNA-LPX) formulated on DOTMA combined with DOPE, can target to deliver the mRNA into dendritic cells (DCs) and macrophages by changing the surface charge ratio from positive to negative through intravenous administration, and induce an antigen-specific adaptive immune response and type-I-IFN-associated innate immune response for cancer immunogenic therapy (Kranz et al., 2016). The same lipid formulation for the treatment of autoimmune encephalomyelitis is utilized as a non-inflammatory mRNA vaccine (Krienke et al., 2021 January 8). The hybrid Poly (lactic-co-glycolic acid) (PLGA)-DOTAP core carrying histone deacetylase inhibitors (HDACIs) mRNA to inhibit metastatic lung cancer was also developed (Peng et al., 2021). Metal−lipid hybrid nanoparticle (MLN) containing EDOPC to mediate genome editing transforming growth factor-β (TGF-β) can restructure the tumor microenvironment and prevent tumor recurrence in combination with near-infrared light to heat (Kim et al., 2021). BHEM-Cholesterol was developed by replacing the methyl group with two hydroxyl regions on the basis of DC-Cholesterol in the head group aimed to augment membrane/lipid interaction, combined with poly(ethylene glycol)-block-poly(lactic-co-glycolic acid) (PEG-b-PLG) and PLGA to synthesize a lipid–polymer hybrid particle for mRNA entrapment (Zeng et al., 2020b).

Cationic lipid-based nanoparticles have shown wide application prospects in mRNA therapy, not only for their capability to promote mRNA molecules to stay in LNPs core but also because they can elicit an innate immune response, thus acting as immune adjuvants to improve immunogenicity (Bevers et al., 2022). For example, DDAB is a cationic lipid as an immune adjuvant that stimulates an immune response, activates macrophages, and binds to antigens. The DOTAP-polymeric hybrid nanoparticles exhibited the most powerful results in inducing humoral and cellular immunity among other candidates *in vivo* (Anderluzzi et al., 2020).

However, we are still facing toxicity issues induced by the permanent positive charge of cationic lipid when broadening the applications of lipid-based non-viral vectors to mRNA therapy, hence, the pH-dependent tertiary amine ionizable lipids that could change to positive charges by protonation effect under lower acidic pH and become electrically neutral at physiological pH were developed to overcome this issue by changing structural domains of cationic compounds. Among fifty-six amino lipids, the most effective LNPs were confirmed with a high acid dissociation constant (pKa) between 6.2 and 6.4 which is close to the protonation point of ionizable lipids (CullisHope, 2017). In an acidified endosome, ionizable amino lipids receive hydrogen ions due to higher pKa than PH. The cationic lipids enhance interaction with negatively charged endosome bilayer and promote the endosome membrane unstableness and endosomal escape, which is a major obstacle in LNP technology, following internalization by the target cell and the release of RNA molecules into the cytosol (Farbiak et al., 2021; Dirisala et al., 2022). Hence, ionizable cationic lipids exhibit the advantage of minor toxicity and prolonged existing times in the body in comparison with cationic delivery vehicles after extracellular vaccination.

Ionizable lipids, such as DOGS, multivalent lipid 5 (MVL5), DC-Cholesterol and GL67, initially get development for DNA transfection, later, some have also been investigated for mRNA therapy. Heyes et al. synthesized DODMA by modifying DOTMA and further displaced the oleyl lipid tails with the linoleyl chains to form DLinDMA, and then compared the effect of different saturation degrees on gene silencing ability (Heyes et al., 2005). Taking linoleyl alcohol as raw material, DLin-KC2-DMA was synthesized through five steps reactions with pKa values between 6.2 and 6.7, and has vastly higher activity than DLinDMA (Semple et al., 2010). In order to screen for ionizable lipids, the Factor VII model offers a high-throughput approach for hepatic gene silencing with effective siRNA dose, and further modifications on the linker domain led to the development of DLin-MC3-DMA (MC3). It has been proven to work effectively in low doses and is a key component of Onpattro, the first siRNA drug approved by the United States Food and Drug Administration (FDA) (Akinc et al., 2019). To improve the biocompatibility of LNPs, the researchers focused on lipid biodegradable functionality by introducing ester groups into the hydrocarbon tail, which can be easily degraded to reduce potential toxicity, immunogenicity, and other side effects. By changing the ester bond position, L319 was proven to be the lead biodegradable tertiary amine lipid (Maier et al., 2013). In the same way, the biodegradable lipids ATX-100, Lipid 5, Lipid H (SM-102), ALC-0315, Acuitas A9 and LP-01 have better delivery ability *in vivo* pharmacokinetics (Buschmann et al., 2021). These results demonstrated that the branching alkyl tails may improve RNA molecules’ release within the endosome. Additionally, Lipid H nanoparticles revealed a relationship between LNPs size and immunogenicity in mice from various formulations (Hassett et al., 2021). Further, A6 incorporating alkyne groups into the non-polar tails could facilitate their interaction with the endosome membrane, thus enhancing endosomal escape from aqueous inner core (Miao et al., 2020). In Lokugamage et al. (2019) synthesized a library of 16 diverse ionizable lipids using diethylamino head region or 1-pyrrodinyl head group conjugated to carbonate linkers and the 11-A-M LNPs perform the best effects than others. The CMVLn (*n* = 2–5), a new degradable multivalent cationic lipids containing a disulfide-bond group between the head group and the hydrophobic tails, and this disulfide bond could be cleaved by reducing agents leading to attenuate toxicity (Shirazi et al., 2011). The ssPalmE, a lipid-like nanomaterial with a disulfide bond and incorporated alpha-tocopherol succinate as the lipid tails, could efficiently suppress tumor growth (Akita et al., 2015). ssPalmO-Phenyl-P4C2, optimized from ssPalmE, using oleic acid as the scaffold of biodegradable lipidoid material showed superior delivery efficiency. Additionally, the introduction of the aromatic ring surprisingly contributes to endosomal membrane destabilization (Tanaka et al., 2020).

The pH-sensitive amino lipid YSK05 displays high fusogenic with the cell membrane (Miyabe et al., 2014). Further optimization of the hydrophilic head region of YSK05 contributed to YSK12, which significantly facilitated gene silencing in mouse DCs (Warashina et al., 2016). The efficient gene silencing ability (50% effective dose: 0.0025 mg/kg) and biodegradability were observed in the third generation YSK series of ionizable lipids CL4H6 (Sato et al., 2019).

In 2014, ionizable truncated acyl backbone amino lipids with asymmetric tails were reported. They were well-tolerated and showed high scavenging capacity *in vivo* addressing a key pharmaceutical challenge through rational formulation optimization (Gindy et al., 2014). Derived from this work, Suzuki et al. (2017) synthesized a string of ionizable lipids with two asymmetric hydrocarbon tails (e.g., L021), and further optimization of ester bond into the lipid tail generated L101, a biodegradable lipid displayed potent gene-silencing activity in mouse hepatocytes and resulted in rapid pharmacokinetics of the lipid.

Combinatorial conjugation and screening approaches have been applied for the synthesis of ionizable lipidoid libraries to improve delivery efficiency. With Design of Experiment methods, several types of reactions have been applied for the synthesis of lipidoids, such as the ring-opening reaction of epoxides, reductive animation reaction and Michael Addition (Zhang et al., 2021; Hu et al., 2022). In 2008, Anderson’s laboratory synthesized a large library of more than 1,200 structurally varied lipid-like materials, and the 98N12-5 acting as the top one performed potent specific gene knockdown in three animal models (Akinc et al., 2008). In addition, they developed two-generation libraries, 304O_13_ and 503O_13_ turned out to be the lead lipids respectively, with high levels of transportation efficiency (Whitehead et al., 2014). O_i10_, a ten-carbon branched tail, was conjugated to alkyl-amine to synthesize 306O_i10_ which can potently ionize at low pH and facilitate more efficient mRNA delivery (Hajj et al., 2019). An ionizable lipid library was synthesized by the addition reaction of amine head and acrylate tail containing the disulfide bond, among which, 306-O12B indicated the capability of liver targeting through co-delivery of Cas9 mRNA and gRNA for Angptl3 gene editing *in vivo* (Qiu et al., 2021). BAMEA-O16B, a biodegradable lipid integrated with disulfide bonds, can simultaneously deliver Cas9 mRNA and sgRNA into cells for gene editing (Liu et al., 2019). A library of over 1,500 bio-reducible lipidoids was constructed *via* addition between diverse amine groups and acrylate esters reacted with alkyl thiols. 5A2-SC8, the lead-performing lipid of the library, provided a potent therapeutic material for gene therapy system (Zhou et al., 2016). Based on 5A2-SC8, replacing amino scaffold 5A2 with 4A3 results in 4A3-SC8, which was formulated in dendrimer-based lipid nanoparticles and achieved gene editing through CRISPR/Cas9 protein (Farbiak et al., 2021 July). Anderson’s group synthesized a lipidoid library *via* another ring-opening approach of epoxides altering carbon lengths with amine substances, such as C12-200 and cKK-E12. A1-D1-5 also called iLY 1809, a thermostable analog of cKK-E12, achieved an effective dose of 0.18 mg/kg of ED_50_ for siRNA (Hu et al., 2022) and enable robust gene editing *via* the delivery of plasmid which expresses both Cas9 protein and sgRNA targeted for Polo-like kinase 1 (PLK1) (Li et al., 2022). Additionally, 7C1 (Lokugamage et al., 2021) and G0-C14 (Islam et al., 2021) were synthesized *via* ring-opening of alkyl epoxide with PEI600 and generation 0 of poly (amidoamine) (PAMAM) respectively.

Amines, isocyanides, and alkyl ketones were used to synthesize a library of 1,080 ionizable lipids. The high-performing lipids, A18-Iso5-2DC18 can induce a strong immune response and show remarkable tumor growth suppression and the survival was prolonged in tumor models through the systematic administration of LNPs (Miao et al., 2019). Lipid-like derivatives (TT2-TT8) were developed *via* Reductive Amination. TT3-LNP can potently provide protection for human factor IX mRNA (Li et al., 2015), and in 2020, it was used to encapsulate the SARS-CoV-2 spike subunits mRNA (Zeng et al., 2020a). FTT5, a biodegradable analog of TT3, further facilitates the release of mRNA encoding human factor VIII into cytoplasm (Zhang et al., 2020). Most recently, Cornebise et al. (2022) reported that squaramides as a part of aromatic four-membered rings, participated in pi-pi interactions, hence, the top-performing lipid 29 can cause high expression of mRNA.

### Helper lipids—phospholipid, cholesterol and PEGylating lipids

**FIGURE 6:**
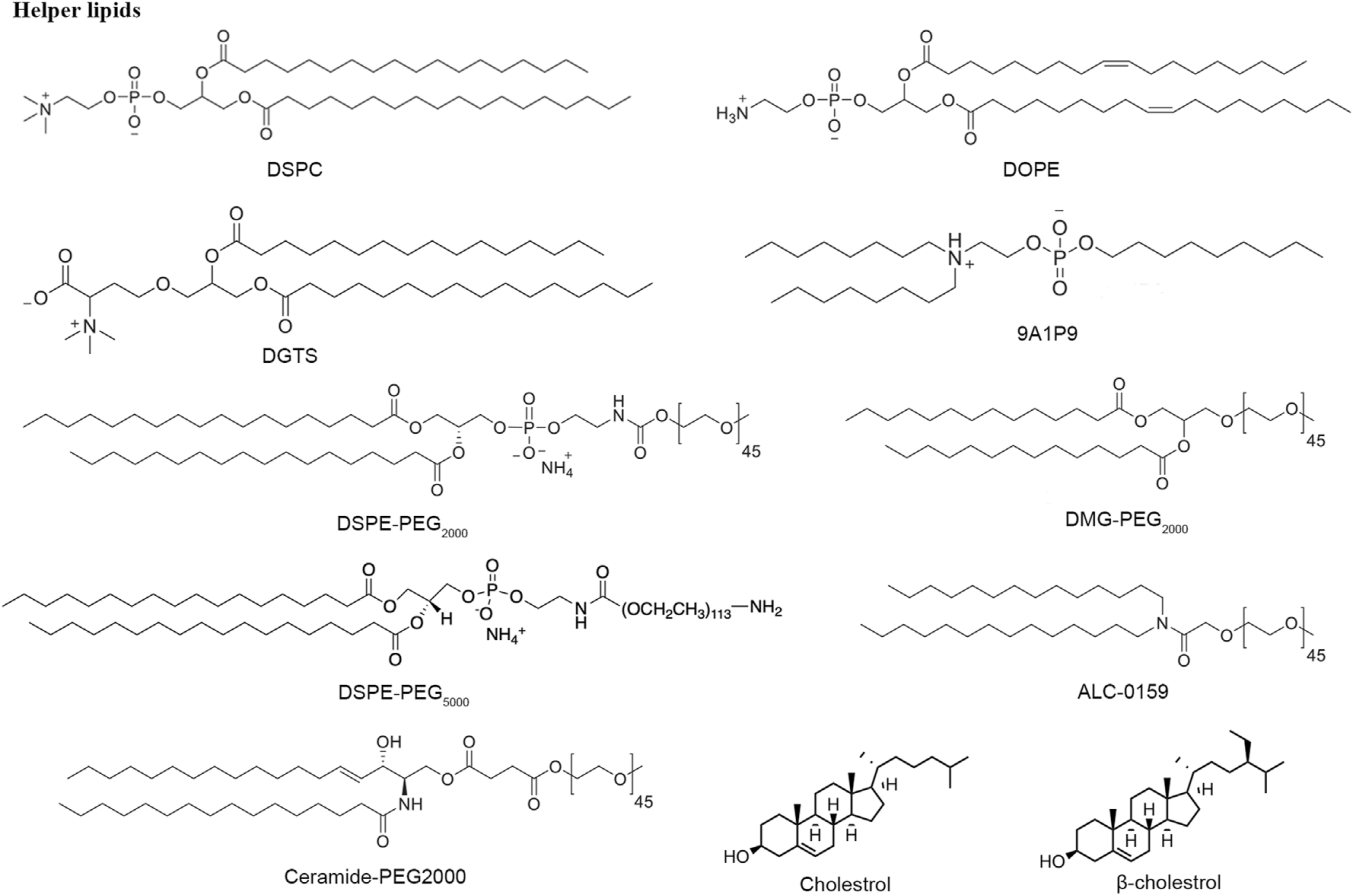
Structure of some helper lipids.

Extensive research has shown that LNP generally consists of additional lipid nanomaterials besides cationic or ionizable lipids to stabilize the RNA delivery system, such as phospholipids, cholesterol, and polyethylene glycol lipids (Figure 6). Phosphatidylcholines (PCs), characterized by cylindrical geometry structure, is the most abundant phospholipid of cell membrane bilayers, approximately accounting for about 50%. DSPC has two symmetric saturated tails with high melting temperatures and is typically used to formulate highly stable LNPs (Witzigmann et al., 2020). Recently, due to the pandemic of COVID-19, DSPC has been applied for mRNA-1273 and BNT162b2 vaccines (Puranik et al., 2022). DOPE, a phosphoethanolamine with two unsaturated tails groups, replacing DSPC in LNPs formulations achieves potent mRNA delivery efficiency *in vivo* because DOPE is inclined to adopt a hexagonal H(II) conformation transformation, which makes endosome bilayer more unstable and accelerates endosome escape of lipid nanoparticles after uptake (Hou et al., 2021). The 9A1P9 from a library of ionizable phospholipids through combinatorial reaction schemes got 40 and 965 times more efficient than DOPE and DSPC in mRNA delivery, respectively (Liu et al., 2021).

As a key member of the lipid nanoparticle formulation, cholesterol can serve as a helper lipid that can make nanoparticles more stable by filling in gaps between phospholipids and facilitating the fusion of the nanoparticles with the biological membrane (Zhang et al., 2021). The studies reported that the amount of cholesterol in LNP components is larger than what can be stably retained in LNPs. Moreover, it was demonstrated that about 30–40 mol% helper lipid is necessary for the encapsulation of siRNA into LNPs, providing another insight into the part of helper lipids.

The enhanced permeability and retention (EPR) effect influences the preferential accumulation of LNP into solid tumors (Sindhwani et al., 2020; Zheng and LiDing., 2021). However, this effect would depend on relatively longer circulation time and relatively small particle size. Liu et al. (2022) synthesized a new shielding material by conjugating low molecular weight polyethylene glycol (PEG) to a hyaluronic acid (HA) core forming a protective layer through electrostatic interactions. Hence, PEGylating lipids are incorporated into LNP formulations to extend the circulation time within the blood by reducing immune clearance regulated by the kidneys. (Forfull names of lipid compounds, see Supplementary Material).

## Potent cancer treatment

International Agency for Research on Cancer (IRAC) reported that there are 19,292,789 new cancer cases and 9,958,133 cancer deaths all over the world with 40 types in 2020 and the top three are breast, lung, and colorectal cancer. Therefore, it is critical to develop the anti-tumor vaccine. More recently, Moderna and Pfizer/BioNTech LNP-mRNA vaccines approved by the FDA to prevent the pandemic of COVID-19, are receiving global attention. The main application associated with the use of LNP for cancer therapy has been broadly studied during the past years, and still, nanoparticles display potent interest because of their relatively small size and large surface-to-volume ratio, and they lead to absolutely novel revolution compared to the large vehicles of bulk material. Besides, LNP-mRNA vaccines have several advantages over conventional vaccines: 1) safety, they eliminate concerns associated with intracellular toxicity and infection risk (CullisHope, 2017). In contrast with DNA nucleoside vaccines and viral vehicle-based vaccines, the risk of genomic integration and toxicity is not posed in mRNA vaccines because they act in their activity in the cytoplasm. The transient property of mRNA molecules is beneficial to avoiding an overexpression of the target protein. Additionally, the researchers have developed a procession of favorable degradable biomaterials. 2) flexibility, mRNA vaccines can easily achieve different targets by changing the coding region and furthermore enable the co-delivery of multiple mRNA molecules to the same cell allowing the generation of multi-antigen complexes thus we can simultaneously prevent different pathogens by injecting a single vaccine. 3) Speediness and repeatability, GMP-leveled manufacture of mRNA vaccines enable reproducible and homogeneous production of lipid-mRNA nanoparticles within weeks (Gebre et al., 2021).

### Immunotherapy

**FIGURE 7:**
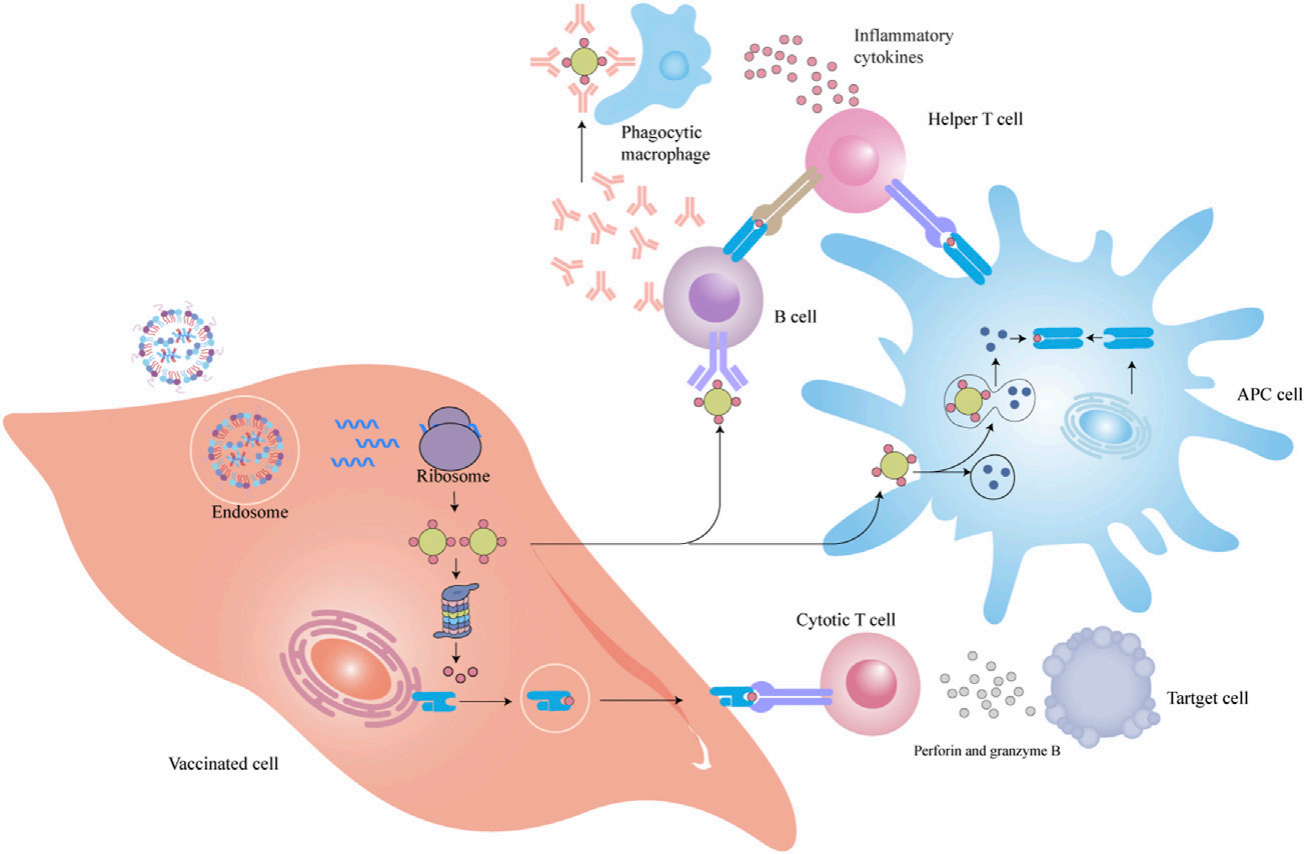
LNP-mRNA vaccines immune response process.

The majority of mRNA-LNP vaccines can be internalized by the uptake of vaccinated cells through systemic administration and can express into antigen proteins that are presented to T and B cells by MHC molecules to kill diseased cells and facilitate the death of tumor cells (Figure 7).

Cancer vaccines involve the elicitation of tumor-specific T lymphocyte response aiming at removing or rejecting tumor cells without harming normal cells by providing cancer antigens under the action of immune stimulators. The attempt to use mRNA to treat tumors can date back to 1995, which showed to elicit antigen-specific immune responses in mice (Hou et al., 2021). Prior to the broad application of the LNP-mRNA anti-cancer vaccine, one of the key manufacturing limitations is that it is difficult to select a target antigen. Following the discovery of Tumor-associated antigens (TAAs), we can design cancer vaccines to target TAAs that are preferentially translated into malignant cells. The proof-of-concept research of LNPs from 2016 by Kranz et al. reported that the RNA-LPX vaccine can target to DCs *in vivo via* intravenous injection of TAAs-lipoplexes based on DOTMA or DOTAP cationic lipid combined with DOPE (Kranz et al., 2016). The encapsulation of TAAs-mRNA into the lipoplex efficiently kept the TAAs molecules from extracellular ribonucleases and boosted their expression in various lymphoid organs. And the authors demonstrated that not only can the mRNA-lipoplex vaccine elicit a type I IFN-associated innate immune response, but also lead to a potent adaptive response, efficiently down-regulating the growth of tumors. DC-based immunotherapies make up the main part of mRNA cancer vaccines in clinical trials and delivery of mRNA molecules into DCs for adoptive immune was the first-in-human clinical trial based on an mRNA therapeutic cancer vaccine (Kowalski et al., 2019). And currently, lipid nanoparticle–mRNA formulations for cancer are extensively developed as a consequence of the promising antitumor efficacy shown in CureVac, BioNTech, and Moderna as pioneers in the battle.

To further improve vaccine effects, we can optimize the processes of APC uptake and T cell activation by co-delivery of antigen mRNA cargos with adjuvants and co-stimulatory molecules to obtain a more comprehensive oncology therapeutic effect, for instance, mannose functionalized LNPs vaccines are preferentially taken up by DCs (Van der Jeught et al., 2018). Stimulator of interferon genes (STING) is key receptor proteins in the natural immune response that can co-adjust innate immunity and acquired immunity. After vaccination, STING will evoke type I interferon. The occurrence of cytokines and T lymph cell recruiting factors arouse tumor-specific cytotoxic T cells (CTLs) response, which can accelerate the expression of antigen *in vivo*, as well as evoke the STING signaling pathway, and remarkably promote the tumor-associated antigen immune activation (Li et al., 2022). At the same moment, it is worth noting that T cell initiation may better determine whether an immune response will be elicited rather than antigen expression as the final criteria for assessing the efficiency of an mRNA vaccine. Another mRNA-2752 involves OX40L/IL-23/IL-36 multiple mRNA macromolecules to treat lymphoma (NCT03739931). Within the mRNA-2752 cocktail vaccine, OX40L functions as the activated co-stimulating signal to potentiate T cell effector function and expansion. IL-36 acts as pro-inflammatory cytokines to further augment anticancer effects and elicit a favorable tumor microenvironments (TME) change. IL-23, as a member of the IL-12 family, functions both in innate immunity and adaptive immunity (Hewitt et al., 2019).

With the recent advancement in cancer immunotherapies, the application of immune checkpoint inhibitors, particularly the discovery of neo-antigens and the development of personalized vaccines, a series of improvements have been achieved to reveal the feasibility of mRNA vaccines to conquer cancer. Several immune checkpoint blockades, monoclonal antibodies (mAbs) targeting immune regulatory molecules have been found out in the last few years, such as anti-programmed cell death 1 (PD-1), programmed cell death-ligand 1 (PD-L1) and cytotoxic T-lymphocyte-associated protein 4 inhibitors. FixVac melanoma mRNA vaccine formulated with four non-mutated antigens combined with a checkpoint inhibitor, anti-PD-1 antibody mediates persistent immune responses in patients (Sahin et al., 2020). Wu et al. formulated IVT-mRNA encoding the Pembrolizumab, a commercial anti-PD-1 mAb in LNP *via* optimizing the addition of signal peptide and the molar ratio of heavy/light chain of antibody leading to an excellent tumor growth inhibition (Wu et al., 2022). Tumor-associated macrophages (TAMs) are extremely abundant and play an important part in provoking immunosuppression in liver malignancies. To eliminate this immunosuppression, an antibody that specifically neutralizes CCL2 and CCL5 (BisCCL2/5i), two key chemokines taking the responsibility for TAM infiltration, is developed. And BisCCL2/5i combined with PD-1 ligand inhibitor encapsulating in lipid nanoparticles leads to death inhibition in mouse models of primary or metastasizing liver cancer (Wang et al., 2021).

Cancer neo-antigens originated from non-synonymous somatic mutations in tumor cells represent a promising orientation for cancer immunotherapies and neo-antigen-based cancer vaccines currently exhibit prominent therapeutic capacity in both preclinical and clinical trials (Miao et al., 2021). The mRNA-4157 is a personalized therapeutic vaccine for melanoma from Moderna. An attractive therapeutic candidate approach that combined a novel CpG-B class oligodeoxynucleotide (CpG 2018B) enhanced cytokine generation with neo-antigen mRNA and promoted the antitumor effect *via* intratumoral injection (Li et al., 2021). Other recent work has paid attention to chimeric antigen receptor (CAR)-T-cell cancer immunotherapy. Specifically, synthetic mRNA molecules encoding CAR are internalized into T lymph cells, and then CAR protein scaffolds are expressed in T cells to identify and kill antigen-specific tumor cells (Gao et al., 2021). Ionizable lipids have delivered CAR mRNA to primary human T cells and achieved potent cancer-killing activity (Billingsley et al., 2020). To overwhelm the limitation of inefficient CAR-T cell stimulation *in vivo*, tight junction protein claudin 6 mRNA composed in RNA-LPX was delivered into lymphoid compartments and promoted the expansion of CAR-T cells in solid tumors (Reinhard et al., 2020). Margaret M. et al. synthesized sequential libraries of ionizable lipid harnessing Orthogonal Design of Experiments for mRNA engineering of CAR-T cells and achieved a 3-fold increase for mRNA expression and low cytotoxicity in comparison to a standard formulation (Billingsley et al., 2022).

### Gene targeting therapy

mRNA therapeutics utilizing LNPs as a targeting delivery vehicle have provided a toolbox in disease states where gene mutations lead to abnormal and non-functional protein expression. In addition to direct delivery of the mutant mRNA to restore function, gene editing such as CRISPR/Cas9 technique served as the next promising application of mRNA therapeutics has been investigated to inactivate or correct mutated cancer-related genes (QiuLi, 2021). However, delivery of the CRISPR/Cas system to malignant tumors for efficient treatment keeps challenging and faces critical obstacles. Wang’s laboratory published the report of hyperbranched poly (β-amino ester) based polyplex nanoparticles involving delivery of CRISPR/Cas9 and sgE7 targeted plasmid and achieved the treatment of HPV infection-associated cervical malignancy (Xiong et al., 2022). Zhang et al. utilized dLNPs for the co-delivery of focal adhesion kinase (FAK) siRNA, Cas9 mRNA and sgRNA-PD-L1 (siFAK + CRISPR-LNPs) to enhance >10-fold gene-editing efficacy in tumor tissues. FAK-knockdown promoted cellular uptake and tumor EPR and CRISPR-LNPs significantly inhibited PD-L1 expression *via* CRISPR/Cas gene editing systems, which achieved a strong tumor growth inhibition and metastasis in four different mouse models of cancer (Zhang et al., 2022). Peer’s laboratory synthesized a novel ionizable amino lipid involving co-delivery of Cas9 mRNA and sgRNAs-PLK1 (sgPLK1-cLNPs) to inhibit tumor growth and prolong survival by 80% (Rosenblum et al., 2020). Recently, TME, which play a critical role in activating immune cells, has gained attention for immunotherapy. TGF-β, a major immunosuppressive cytokine, widely interacts with various immune cells and can induce immune evasion of the TME. Kim et al. successfully used MLN encapsulating sgRNA targeting TGF-β and CRISPR/Cas9 system to restructure the TME and protect against tumor recurrence (Kim et al., 2021). The ability of gene editing to disrupt gene expression in tumors opens a new era for cancer treatment. And more research is desired to explore the next-generation nano-vectors to deliver the CRISPR/Cas9 mRNA genome editing tool to treat cancers for clinical application.

## Summary and future prospective

LNP-based mRNA therapies have retained great appeal and a promising rank of drugs for diversified therapeutic applications were developed over the past few years by replacing ideal mRNA cargoes. Importantly, BNT162b2 (Pfizer/BioNTech) raises robust prevention for the COVID-19 pandemic and a new era of mRNA therapeutics has been opened up. Although significant advances have been achieved in recent years, it is worth noting that several obstacles still hamper the broad applications of LNP-mRNA therapies. It is a long road to optimize nonviral vector formulations for nucleic acid delivery, which the development of Patisiran perhaps best exemplified. Meanwhile, LNP-mRNA development has submitted fundamental insights into optimal formulations for delivery efficiency from endosome escape, GMP, safety profiles, stability, and cost-effectiveness to establish next-generation mRNA-LNPs.

At the same moment, the determinants of performance for LNP-mRNA delivery technology are multifactorial and interactive and they bear a versatile toolbox with many possibilities to promote future gene therapies. Future mRNA therapeutic platforms remain to be explored in the following directions: 1) selective organ targeting (SORT) technology needed to be detailed in the LNP corona composition 2) personalized framework 3) reprogramming the tumor microenvironment, to promote CD4^+^ and CD8^+^ cells infiltrates in the tumors 4) biodegradablity and biocompatibility, covalently coupling degradable chemical groups to reduce cytotoxicity 5) pharmacokinetics and pharmacodynamics, appropriate duration of pharmacological effects, and 6) adjuvanticity, the relationship between innate immune and inflammation. As these design criteria and modular parameters are rigorously investigated and established with large multidimensional spaces, it is anticipated that a therapeutic revolution that breaks through the current limits will come in the near future.
