# Supplementary material for: Lipid-mRNA nanoparticles landscape for cancer therapy
Source: Front Bioeng Biotechnol. 2022 Oct 31;10:1053197. doi: 10.3389/fbioe.2022.1053197 (PMC9659646; doi:10.3389/fbioe.2022.1053197)
Supplement: Supplementary file 1 [file Table1.DOCX]

**Lipid-mRNA nanoparticles landscape for cancer therapy**

Yin Li ^&,1^, Hengtong Fang^&,1^, Tao Zhang^1^, Yu Wang^1^, Tingting Yu^1^, Bai Li**^*^**^,2^, Huping Jiao**^*^**^,1^.

^1^College of Animal Science, Jilin University, Changchun, Jilin Province, People’s Republic of China

^2^ Department of Colorectal &Anal Surgery, General Surgery Center, First Hospital of Jilin University, Changchun, Jilin Province, People’s Republic of China

^&^ These authors contributed equally to this work.

***** Correspondence should be addressed to Huping Jiao (jiaohp@jlu.edu.cn ) or Bai Li(bai-li@jlu.edu.cn) College of Animal Science, Jilin University, 5333 Xian Road, Lvyuan District, Changchun 130062, Jilin Province, People’s Republic of China.

**Supplement Table 1. List of lipids and lipid derivatives names**

| **Abbreviation** | **Full name** |
| --- | --- |
| DOTMA | N-[1-(2,3-dioleyloxy)propyl]-N,N,N-trimethylammonium chloride |
| DOTAP | 1,2-dioleoyl-3- trimethylammonium propane |
| DOSPA | 2,3-dioleyloxy-N-[2-(sperminecarboxamido)ethyl]-N,N-dimethyl-1-propanaminium trifluoroacetate |
| EDOPC | 2-[2,3-bis[[(Z)-octadec-9-enoyl]oxy]propoxyethoxyphosphoryl]oxyethyl-trimethylazanium |
| DOPE | 1, 2-dioleoyl-sn-glycero-3-phosphoethanolamine |
| BHEM-Cholesterol | N-bis(2-hydroxyethyl)-N-methyl-N-(2-cholesteryloxy-carbonylaminoethyl) ammonium bromide |
| DC-Cholesterol | 3β-[N-(N’,N’-dimethylaminoethane)-carbamoyl]-cholesterol |
| DDAB | N-decyl-N,N-dimethyldecan-1-aminium bromide |
| DOGS | (2S)-2,5-bis(3-aminopropylamino)-N-[2-(dioctadecylamino)acetyl]-pentanamide |
| multivalent lipid 5(MVL5) | N^1^-[2-((1S)-1-[(3-aminopropyl)amino]-4-[di(3-aminopropyl)amino]-butylcarboxamido)ethyl]-3,4-di[oleyloxy]-benzamide |
| GL67 | 10,13-dimethyl-17-(6-methylheptan-2-yl)-2,3,4,7,8,9,10,11,12,13,14,15,16,17-tetradecahydro-1H-cyclopenta[a]phenanthren-3-yl(3-aminopropyl)(4-((3-aminopropyl)amino)butyl)carbamate |
| DODAP | 1,2-dioleoyl-3-dimethylammonium-propane |
| DODMA | 1,2-dioleyloxy-3-dimethylaminopropane |
| DLinDMA | 1,2-dilinoleyloxy-3-dimethylaminopropane |
| DLin-KC2-DMA | 2,2-dilinoleyl-4-dimethylaminoethyl-[1,3]-dioxolane |
| DLin-MC3-DMA; MC3 | (6Z,9Z,28Z,31Z)-heptatriacont-6,9,28,31-tetraene-19-yl4-(dimethylamino)butanoate |
| L319 | di((Z)-non-2-en-1-yl)9-((4 (dimethylamino)butanoyl)oxy)-heptadecanedioate |
| ATX-100 | (Z)-Non-2-en-1-yl N-(((2-(Dimethylamino)propyl)thio)carbonyl)-N-(2-oxo-2-(pentadecan-8-yloxy) ethyl)glycinate |
| Lipid 5 | heptadecan-9-yl 8-((2-hydroxyethyl)(8-(nonyloxy)-8 oxooctyl)amino)octanoate |
| Lipid H (SM-102) | heptadecan-9-yl 8-((2-hydroxyethyl)(6-oxo-6 (undecyloxy)hexyl)amino)octanoate |
| ALC-0315 | ((4-hydroxybutyl)azanediyl)bis(hexane-6,1-diyl)bis(2-hexyldecanoate) |
| Acuitas A9 | bis(2-butyldecyl) 10-(N-(3-(dimethylamino)propyl)nonanamido)nonadecanedioate |
| A6 | di(dec-3-yn-1-yl) 9-((4-(dimethylamino)butanoyl)oxy)heptadecanedioate |
| LP-01 | 3-((4,4-bis(octyloxy)butanoyl)oxy)-2-((((3-(diethylamino)propoxy)carbonyl)oxy)methyl)propyl(9Z,12Z)-octadeca-9,12-dienoate |
| 11-A-M | 3-(2-((3r,5r,7r)-adamantan-1-yl)acetoxy)-2-((((3-(diethylamino)propoxy)carbonyl)oxy)methyl)propyl(9Z,12Z)-octadeca-9,12-dienoate |
| CMVL4 | N-(2-(((2S)-2-(2,5-Bis(3-aminopropylamino)pentanamido)ethyl)disulfanyl)ethyl){3,4di(oleyloxy)benz-amide}tetra-trifluoroacetate |
| ssPalmE | O,O'-(((disulfanediylbis(ethane-2,1-diyl))bis(azanediyl))bis(propane-3,1-diyl))bis((R)-2,5,7,8-tetramethyl-2-((4R,8R)-4,8,12 trimethyltridecyl)chroman-6-yl) disuccinate |
| ssPalmO-Phe | (((((disulfanediylbis(ethane-2,1-diyl))bis(piperidine-1,4-diyl))bis(ethane-2,1-diyl))bis(oxy))bis(2-oxoethane-2,1-diyl))bis(4,1-phenylene) dioleate |
| CLD | di((Z)-octadec-9-en-1-yl)3,30-disulfanediylbis-(2-(2,6-diaminohexanamido)propanoate) |
| YSK05 | 1-methyl-4,4-bis(((9Z,12Z)-octadeca-9,12-dien-1-yl)oxy)piperidine |
| YSK12 | (6Z,9Z,28Z,31Z)-19-(4-(dimethylamino)butyl)heptatriaconta-6,9,28,31-tetraen-19-ol |
| CL4H6 | (Z)-7-(4-(dipropylamino)butyl)-7-hydroxydotriacont-23-en-1-yl oleate |
| L021 | 1-(2-octylcyclopropyl)heptadecan-8-yl 1-methylpiperidine-4-carboxylate |
| L101 | (Z)-1-(non-2-en-1-yloxy)-1-oxononadecan-10-yl 1-methylpiperidine-4-carboxylate |
| 98N12-5 | 3,3',3'',3'''-((((2-((3-(dodecylamino)-3-oxopropyl)amino)ethyl)azanediyl)bis(ethane-2,1-diyl))bis(azanetriyl))tetrakis(N-dodecylpropanamide) |
| 304O_13_ | 1,1',1''-((nitrilotris(ethane-2,1-diyl))tris(methylazanediyl))tris(heptadecan-3-one) |
| 503O_13_ | 21,25-dimethyl-18,28-bis(3-oxoheptadecyl)-18,21,25,28-tetraazapentatetracontane-15,31-dione |
| 306O_i10_ | tetrakis(8-methylnonyl) 3,3′,3″,3‴-(((methylazanediyl) bis(propane-3,1-diyl))bis(azanetriyl))tetrapropionate |
| 306O-12B | tetrakis(2-(octyldisulfaneyl)ethyl) 3,3',3'',3'''-(((methylazanediyl)bis(propane-3,1-diyl))bis(azanetriyl))tetrapropionate |
| BAMEA-O16B | bis(2-(dodecyldisulfaneyl)ethyl) 3,3'-((3-methyl-9-oxo-10-oxa-13,14-dithia-3,6-diazahexacosyl)azanediyl)dipropionate |
| 5A2-SC8 | bis(2-((2-methyl-3-(octylthio)propanoyl)oxy)ethyl)4,10,16-tris(3-(2-((2-methyl-3-(octylthio)propanoyl)oxy)ethoxy)-3-oxopropyl)-4,7,10,13,16-pentaazanonadecanedioate |
| C12-200 | 1,1′-((2-(4-(2-((2-(bis(2- hydroxydodecyl)amino)ethyl) (2-hydroxydodecyl)amino)ethyl)piperazin-1-yl)ethyl)azanediyl) bis(dodecan-2-ol) |
| cKK-E12 | 3,6-bis(4-(bis(2-hydroxydodecyl)amino)butyl)piperazine-2,5-dione |
| A1-D1-5 (iLY1809) | N^1^, N^4^-bis(3-(bis(2-hydroxydodecyl)amino)propyl)succinimide |
| OF-02 | 3,6-bis(4-(bis((9Z,12Z)-2- hydroxyoctadeca-9,12-dien-1-yl)amino)butyl)piperazine-2,5-dione |
| OF- Deg- Lin | (((3,6-dioxopiperazine-2,5-diyl)bis(butane-4,1- diyl))bis(azanetriyl))tetrakis(ethane-2,1-diyl) (9Z,9′Z,9″Z,9‴Z,12Z,12′Z,12″Z,12‴Z)-tetrakis (octadeca-9,12-dienoate) |
| OF-C4-Deg-Lin | (((3,6-dioxopiperazine-2,5-diyl)bis(butane-4,1-diyl))bis(azanetriyl))tetrakis (butane-4,1-diyl) (9Z,9′Z,9″Z,9‴Z,12Z,12′Z,12″Z,12‴Z)-tetrakis (octadeca-9,12-dienoate) |
| 7C1 | 19,31-bis(2-aminoethyl)-28-(2-((2-aminoethyl)amino)ethyl)-25-(2-((2-((2-hydroxypentadecyl)amino)ethyl)amino)ethyl)-16,19,22,25,28,31,36-heptaazahenpentacontane-14,38-diol |
| G0-C14 | 3,3'-(13,34-dihydroxy-15,32-bis(2-hydroxytetradecyl)-19,28-dioxo-15,18,22,25,29,32-hexaazahexatetracontane-22,25-diyl)bis(N-(2-((2-hydroxytetradecyl)amino)ethyl)propanamide) |
| A18-Iso5-2DC18 | ethyl 1-(3-(2-ethylpiperidin-1-yl)propyl)-5,5-di((Z)-heptadec-8-en-1-yl)-2,5-dihydro-1H-imidazole-2-carboxylate |
| TT3 | *N^1^,N^3^,N^5^*-tris(3-(didodecylamino)propyl)benzene-1,3,5-tricarboxamide |
| FTT5 | Hexa(octan-3-yl) 9,9′,9″,9‴,9″″,9‴″-((((benzene-1,3,5- tricarbonyl)ris(azanediyl))tris(propane-3,1-diyl))tris(azanetriyl))  hexanonanoate |
| lipid 29 | 3-(methylamino)-4-((3-((10-octyloctadecyl)(8-(undecan-3-yloxy)non-8-en-1-yl)amino)propyl)amino)cyclobutane-1,2-dione |
| XMaN6 | (1s,3s,5s)-*N^1^,N^3^,N^5^*-tris(6-(didodecylamino)hexyl)adamantane-1,3,5-tricarboxamide |
| 9A1P9 | 2-(dioctylammonio)ethyl nonyl phosphate |
| ALC-0159 | 2-(2-methoxyethoxy)-N,N-ditetradecylacetamide |
| DSPC | 1,2-dioctadecanoyl-sn-glycero-3-phosphocholine |
| DOPE | 1,2-dioleoyl-sn-glycero-3-phospho-Ethanolamine |
